# Multi-site performance evaluation of the Alinity m Molecular assay for quantifying Epstein-Barr virus DNA in plasma samples

**DOI:** 10.1128/jcm.00472-23

**Published:** 2023-09-20

**Authors:** Els Wessels, Eliseo Albert, Tom Vreeswijk, Eric C. J. Claas, Estela Giménez, Birgit Reinhardt, Mark M. Sasaki, David Navarro

**Affiliations:** 1 Leiden University Medical Center, Leiden, the Netherlands; 2 Hospital Clinico Universitario de Valencia, Valencia, Spain; 3 Abbott GmbH, Wiesbaden, Germany; 4 Abbott Molecular Inc., Des Plaines, Illinois, USA; Mayo Clinic, Rochester, Minnesota, USA

**Keywords:** nucleic acid amplification test, DNA, transplantation, high-throughput diagnostic assay

## Abstract

Detection and monitoring of acute infection or reactivation of Epstein-Barr virus (EBV) are critical for treatment decision-making and to reduce the risk of EBV-related malignancies and other associated diseases in immunocompromised individuals. The analytical and clinical performance of the Alinity m EBV assay was evaluated at two independent study sites; analytical performance was assessed by evaluating precision with a commercially available 5-member EBV verification panel, while the clinical performance of the Alinity m EBV assay was compared to the RealTi*me* EBV assay and a laboratory-developed test (LDT) as the routine test of record (TOR). Analytical analysis demonstrated standard deviation (SD) between 0.08 and 0.13 Log IU/mL. A total of 300 remnant plasma specimens were retested with the Alinity m EBV assay, and results were compared to those of the TOR at the respective study sites (*n* = 148 with the RealTi*m*e EBV assay and *n* = 152 with the LDT EBV assay). Agreement between Alinity m EBV and RealTi*m*e EBV or LDT EBV assays had kappa values of 0.88 and 0.84, respectively, with correlation coefficients *r* of 0.956 and 0.912, while the corresponding observed mean bias was −0.02 and −0.19 Log IU/mL. The Alinity m EBV assay had a short median onboard turnaround time of 2:40 h. Thus, the Alinity m system can shorten the time to results and, therefore, to therapy.

## INTRODUCTION

Approximately 90% of the global population harbors latent Epstein-Barr virus (EBV), a member of the herpesvirus family (1). EBV infection early in life is typically asymptomatic but can cause infectious mononucleosis in adolescents and adults (2). After the initial infection, EBV becomes latent in B lymphocytes. Reactivation of latent virus is associated with various malignancies and autoimmune diseases (3). EBV infection is also a causal factor in the development of post-transplant lymphoproliferative disorders following solid organ or hematopoietic stem cell transplantation. An acute EBV infection or reactivation can present with symptoms similar to those of other viral infections; thus, accurate diagnosis is critical for patient management (4). Longitudinal monitoring of EBV DNA can help evaluate treatment response and detect early EBV reactivation, particularly in immunocompromised patients for whom serological testing may be less accurate (4 – 6).

With PCR being the method of choice in the early diagnosis of a variety of EBV-associated diseases and in monitoring the efficacy of therapies (3), there is an increasing need for reliable, automated EBV assays on consolidated high-throughput platforms to substitute manual or semi-automated commercial assays or laboratory-developed tests (LDTs) with long turnaround times (TAT). This trend is reinforced by the increasing regulation of test procedures within the framework of the new European *In Vitro* Diagnostic Regulation (7).

The Alinity m EBV assay (Abbott Molecular Inc., Des Plaines, IL, USA) (8) is a quantitative PCR assay that is run on the fully automated, continuous, random-access Alinity m analyzer. It has a processing capacity of 300 samples in approximately 8 h and a time to first result of less than 2 h. In this study, analytical, clinical, and TAT performance of the Alinity m EBV assay were assessed.

## MATERIALS AND METHODS

### Molecular EBV assays

The performance of the Alinity m EBV assay was evaluated at two independent International Standard Organization (ISO)-accredited clinical laboratories at the Hospital Clinico Universitario de Valencia, Valencia, Spain, and Leiden University Medical Center, Leiden, the Netherlands. Both study sites used the same lot of Alinity m EBV reagents (amplification, controls, calibrators).

The Alinity m EBV assay is a dual-target (GP350 and EBNA1) dual-probe assay with a sample input volume of 500 µL and a quantitative range from 1.30 to 8.30 Log IU/mL. Alinity m EBV testing was performed at both study sites.

The RealTi*m*e EBV assay (Abbott Molecular Inc., Des Plaines, IL, USA) (9) is a single-target (GP350) PCR test that is run on the *m*2000 platform (Abbott Molecular Inc., Des Plaines, IL, USA). It includes automated sample preparation on the *m*2000sp and PCR amplification and detection on *m*2000rt. The assay uses 500 µL sample input volume and has a quantitative range from 1.60 to 8.30 Log IU/mL. Its clinical performance and utility were previously shown (10). RealTi*m*e EBV testing was performed at the Hospital Clinico Universitario de Valencia, Valencia, Spain.

The LDT EBV assay is based on a previously published PCR assay (11, 12) utilizing primers and a probe to amplify and detect a 74 bp fragment from the EBV BNRF gene, with minor modifications. DNA isolation was performed using 200 µL sample input volume on the MagNA Pure 96 platform with the DNA and Viral NA Small Volume Kit 2.0 and the Pathogen Universal 200 protocol (Roche Diagnostics, Almere, the Netherlands). The PCR was carried out using the HotStar Taq master mix (Qiagen, Hilden, Germany) in the CFX96 real-time detection system (Bio-Rad, Veenendaal, the Netherlands). The assay is standardized to the first WHO International Standard (IS) (13) and quantifies results between 1.0 and 8.0 Log IU/mL. Since in our clinical routine setting, DNA loads <250 IU/mL are not considered clinically relevant, results in this range are reported as <2.4 Log IU/mL. The clinical utility of LDT EBV has been established by its long-term clinical use for nearly 20 years, and the assay was performed at Leiden University Medical Center, Leiden, the Netherlands.

### Alinity m EBV assay analytical performance analysis

To assess the analytical precision of the Alinity m EBV assay, a 5-member EBV verification panel (Exact Diagnostics, Fort Worth, TX, USA) was used containing non-infectious intact whole virus at 2.7–6.7 Log IU/mL in EDTA plasma. Three replicates per panel member were tested over 5 days.

High-positive (HPC) and low-positive (LPC) quality controls were evaluated across the testing sites to assess the reproducibility of the Alinity m EBV assay.

### Clinical specimens

At the two study sites, the performance of the Alinity m EBV assay was compared to the respective comparator EBV assay as TOR.

The study was performed in accordance with the principles of Good Clinical Practice and conducted in adherence with the Declaration of Helsinki. Only remnant patient plasma specimens were used for this study. All clinical specimens were anonymized before study initiation, and an identification number containing no patient identifiers was assigned to each remnant specimen. An approval by an ethics committee was obtained according to the institutional requirement.

Three hundred remnant plasma specimens from either solid organ or hematopoietic stem cell transplant recipients were selected to evaluate the Alinity m EBV assay performance. Specimens were stored at ≤−70°C for up to 6 years. For the study, all specimens were tested using the Alinity m EBV assay, and the results were compared to the historical data that had been obtained by one of the two comparator EBV TORs, respectively, either the RealTi*m*e EBV assay or an LDT EBV assay (Leiden, the Netherlands).

In addition, longitudinal EBV DNA kinetics for 24 patients were compared between Alinity m EBV and LDT EBV. Patients were monitored between 5 and 157 days with a range of 2–13 times.

### Workflow evaluation

For the workflow evaluation on Alinity m, the automatically documented timepoints of loading samples, sample aspiration, and result reporting by the Alinity m instrument were used to evaluate the onboard and processing TATs of the Alinity m system. The onboard TAT was defined as the time interval between loading of the sample racks and reporting of the results while the processing TAT was defined as the time interval between sample aspiration and result reporting.

### Statistical analysis

All analyses were performed using PC SAS (version 9.4) software (SAS, Cary, NC, USA). Relationships between quantitative variables were studied by means of Deming regression. Bland-Altman analysis was performed to evaluate the differences in quantification between the assays.

The following analysis was performed for each instrument and each panel member: The PROC MIXED procedure with the MIVQUE0 option in SAS was used to produce variance components for the model used in the analysis. The point estimates of the means and standard deviations (SD) were reported. The SD was estimated for the within-day component, the between-day component, and the between-site component for each instrument and each panel member. All the effects were considered random for the analyses. Any negative variance components were set to zero for these calculations. The total assay variability was defined as the sum of the within-day (residual error) component, the between-day component, and the between-site component estimates of variability. The following statistics was reported: *N*, mean, within-day SD, between-day SD, between-site SD, Total SD. For the evaluation of the quality controls, a within-day component was not included in the analysis as only one replicate of each HPC and LPC was tested per day and site.

## RESULTS

### Analytical performance

Analytical precision of the Alinity m EBV assay was evaluated across the two study sites by testing a commercially available EBV verification panel consisting of five members ranging in concentration from 2.7 to 6.7 Log IU/mL, running three replicates per panel member over 5 days. As shown in Table 1, the SD measured for each level tested was less than or equal to 0.13 Log IU/mL. The mean difference between the observed values and the target values was −0.33 Log IU/mL indicating a slightly lower quantitation by Alinity m EBV compared to the assigned target value. Reproducibility was characterized by a total SD of 0.05 Log IU/mL for the HPC and of 0.07 Log IU/mL for the LPC (Table 2).

**TABLE 1:** Precision of the Alinity m EBV assay using EBV verification panels (*n* = 30 replicates) tested across two laboratories

Panel member	*N*	Target conc. (Log IU/mL)	Mean conc. (Log IU/mL)	Difference mean-target (Log IU/mL)	Within-day component	Between-day component	Between-site component	Total
					SD (Log IU/mL)	SD (Log IU/mL)	SD (Log IU/mL)	SD (Log IU/mL)
1	30	2.70	2.33	−0.37	0.05	0.00	0.12	0.13
2	30	3.70	3.38	−0.32	0.07	0.02	0.11	0.13
3	30	4.70	4.35	−0.35	0.04	0.03	0.10	0.11
4	30	5.70	5.40	−0.30	0.03	0.01	0.08	0.08
5	30	6.70	6.39	−0.31	0.04	0.03	0.06	0.08

**TABLE 2:** Reproducibility of testing Alinity m EBV quality controls (*n* = 10) across two laboratories

Panel member	*N*	Target conc. (Log IU/mL)	Mean conc. (Log IU/mL)	Difference mean-target (Log IU/mL)	Between-day component	Between-site component	Total
					SD (Log IU/mL)	SD (Log IU/mL)	SD (Log IU/mL)
HPC^*a*^	10	4.80	4.83	0.03	0.03	0.04	0.05
LPC^*b*^	10	2.76	2.79	0.03	0.05	0.04	0.07

^*a*^ HPC, high-positive control.

^*b*^ LPC, low-positive control.

### Clinical performance

A total of 300 clinical plasma specimens were tested with the Alinity m EBV assay. Results were compared to the historical data obtained with the comparator TOR used at the respective study site: the RealTi*m*e EBV assay (*n* = 148 specimens) or an LDT EBV assay (*n* = 152 specimens).

**Fig 1:**
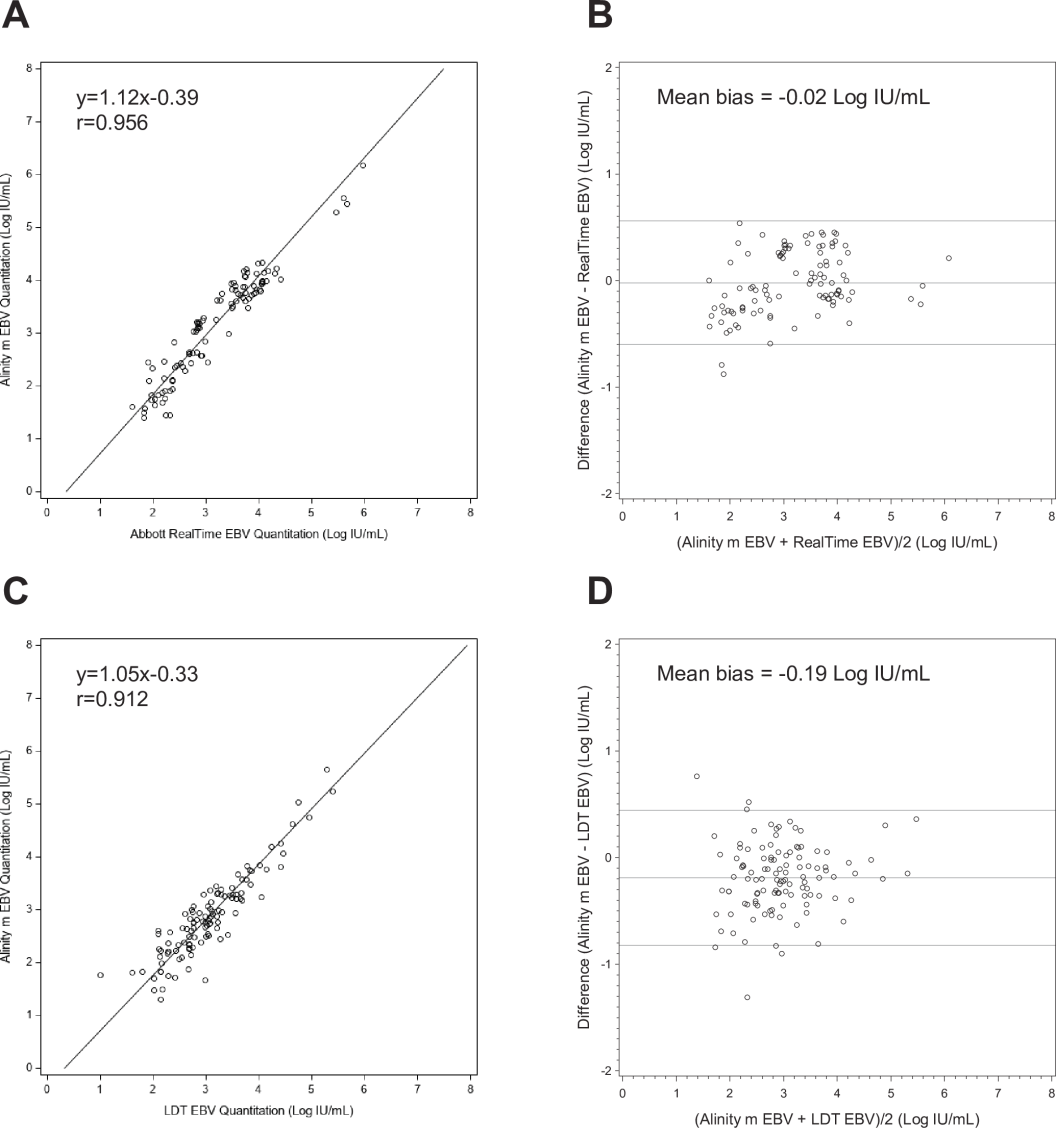
Comparison of the Alinity m EBV assay and test of record (TOR) EBV assay performance with clinical plasma specimens. Deming regression of EBV levels showing the correlation between the Alinity m EBV assay and (**A**) RealTi*m*e EBV assay or (**C**) LDT EBV assay. Bland-Altman analysis shows a mean bias between the Alinity m EBV assay and (**B**) RealTi*m*e EBV assay or (**D**) LDT EBV assay. The middle line indicates the mean bias, and the lines above and below indicate ±1.96 × SD.

A total of 148 clinical remnant specimens with previous results by RealTi*m*e EBV assay were retested with the Alinity m EBV assay. The overall observed qualitative agreement, calculated as concordant negative and positive results (<LLOQ or quantitated) between the two assays, was 96.6% (143/148) with a Cohen’s kappa value of 0.88 representing almost perfect agreement (14) (Table 3). Of the 148 specimens tested, 104 fell within the analytical measuring range (AMR) for both assays. The correlation coefficient *r* was 0.956 (Deming regression equation *y* = 1.12*x* – 0.39, Fig. 1A), and the observed mean bias was −0.02 Log IU/mL (Bland Altman analysis, Alinity m – RealTi*m*e, Fig. 1B).

**TABLE 3:** Agreement of results determined by Alinity m EBV and by RealTi*m*e EBV assays in clinical plasma specimens

		RealTi*m*e EBV				
		Not detected		<LLOQ	Quantitated	Total
Alinity m EBV	Not detected	23		4	0	27
	<LLOQ	1		10	2^*a*^	13
	Quantitated	0		4*^b^*	104	108
	Total	24		18	106	148

^*a*^ Two specimens <LLOQ by Alinity m EBV were quantitated at 1.63 and 1.74 Log IU/mL with the RealTi*m*e EBV assay.

^*b*^ Four specimens <LLOQ by the RealTi*m*e EBV assay had a range of 1.32–1.75 Log IU/mL with the Alinity m EBV assay.

The overall observed qualitative agreement between clinical specimens run on both the Alinity m EBV assay and the LDT EBV assay was 95.4% (145/152) with a Cohen’s kappa value of 0.84 representing almost perfect agreement (Table 4). Of the 152 specimens tested, 113 fell within the AMR of both assays. The correlation coefficient *r* was 0.912 (Deming regression equation *y* = 1.05*x* – 0.33, Fig. 1C), and the observed mean bias was −0.19 Log IU/mL (Bland Altman analysis, Alinity m – LDT, Fig. 1D).

**TABLE 4:** Agreement of results determined by Alinity m EBV and by the LDT EBV assay in clinical plasma specimens

		LDT EBV		
		Not detected	Quantitated	Total
Alinity m EBV	Not detected	22	2^*a*^	24
	<LLOQ	4	10^*b*^	14
	Quantitated	1^*c*^	113	114
	Total	27	125	152

^*a*^ Two specimens Not detected by Alinity m EBV were quantitated at 1.68 and 2.36 Log IU/mL by the LDT EBV assay.

^*b*^ Ten specimens <LLOQ by Alinity m EBV had a range of 1.32–2.25 Log IU/mL with the LDT EBV assay.

^*c*^ One specimen Not detected by LDT EBV was quantitated at 1.67 Log IU/mL with the Alinity m EBV assay.

**Fig 2:**
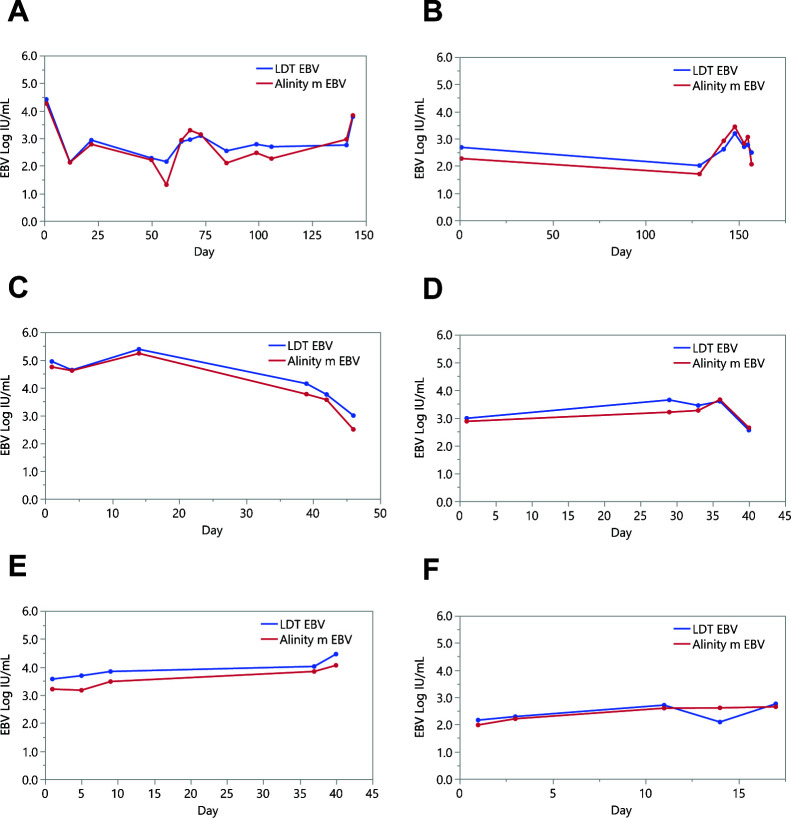
Comparison of EBV DNA kinetics in longitudinal clinical plasma specimens by Alinity m EBV and LDT EBV. Monitoring of EBV DNA loads is shown for six patients with ≥5 tests. (**A**) Patient 1 tested at day 1, 12, 22, 50, 57, 64, 68, 73, 85, 99, 106, 141, and 144. (**B**) Patient 2 tested at day 1, 129, 142, 148, 153, 155, and 157. (**C**) Patient 3 tested at day 1, 4, 14, 39, 42, and 46. (**D**) Patient 4 tested at day 1, 29, 33, 36, and 40. (**E**) Patient 5 tested at day 1, 5, 9, 37, and 40. (**F**) Patient 6 tested at day 1, 3, 11, 14, and 17.

Ninety-five tests of the above 152 specimens were included in a longitudinal analysis performed on 24 patients. During a monitoring period of up to 157 days, the patients’ EBV DNA was determined for 1–12 times following baseline testing. EBV DNA load kinetics were similar between Alinity m EBV and LDT EBV assays. Patients’ kinetics with at least five time points are shown in Fig. 2A through F.

### Workflow analysis

In contrast to the batchwise testing with the TOR assays, Alinity m enables immediate and random loading and testing of samples as they are delivered to the laboratory. Onboard TAT for the Alinity m EBV assay was defined as the time between loading the specimen on the Alinity m system until result reporting. The median onboard TAT was less than 3 h, i.e., 2 h 40 min with a range of 2 h 8 min to 5 h 17 min. Additionally, processing TAT between sample aspiration and result reporting was 113–116 min, with 97% of the results being reported within 115 min. 

## DISCUSSION

In this multi-site evaluation, the Alinity m EBV assay was assessed in an international study in two independent laboratories. A precision study was performed at both study sites, and different lots of bulk solutions could have potentially introduced variability. Despite these variables, overall precision of the Alinity m EBV assay was high with an SD measure of each level tested of ≤0.13 Log IU/mL across the AMR. On average, the quantitation by Alinity m EBV was 0.33 Log IU/mL lower than the manufacturer-assigned target values of the verification panel. This difference in performance could be due to the acceptance range used for value assignment of the verification panel. Beyond that, Alinity m EBV demonstrated excellent reproducibility for the high and low positive assay quality controls (HPC and LPC) with a total SD of 0.05 and 0.07 Log IU/mL, respectively.

Comparing results of clinical plasma samples, we found excellent correlation of Alinity m EBV with RealTi*m*e EBV and an LDT EBV assay with correlation coefficients of *r* = 0.956 and *r* = 0.912 with Cohen’s kappa values of 0.88 and 0.84, respectively. The mean bias observed between the Alinity m EBV and RealTi*m*e EBV was minimal with −0.02 Log IU/mL. Discordant results between the Alinity m and RealTi*m*e assays were only observed around the lower limit of quantitation, i.e., specimens not detected by one assay showed results <LLOQ by the other assay.

Comparable quantitation was also observed between Alinity m EBV and LDT EBV (mean bias −0.19 Log IU/mL). The majority of discordant results (*n* = 5) were again observed around the LLOQ when EBV was not detected by the LDT EBV assay but quantitated at 1.67 Log IU/mL or <LLOQ with the Alinity m EBV assay. Two discordant specimens not detected by Alinity m EBV had been quantitated at 1.68 and 2.36 Log IU/mL by the LDT EBV assay, respectively. The specimen with the quantitation of 1.68 Log IU/mL could not be further investigated due to insufficient volume; however, its result was near the LLOQ of the LDT EBV. The specimen with the quantitation of 2.36 Log IU/mL had sufficient volume for resolution testing with another method, RealTi*m*e EBV. The result obtained with the RealTi*m*e EBV assay (not detected) confirmed the result obtained with the Alinity m EBV assay. We observed >1 Log IU/mL difference with one quantifiable specimen by both assays showing a result of 2.98 Log IU/mL with LDT EBV compared to 1.67 Log IU/mL with Alinity m EBV. As sufficient volume was available, this sample underwent resolution testing with the RealTi*m*e EBV assay. The result obtained with the RealTi*m*e EBV assay (<1.60 Log IU/mL) again confirmed the Alinity m EBV result. Thus, repeat testing in both cases suggested either sample degradation of the EBV DNA, although a sample stability study performed prior study initiation had demonstrated a minimum bias after storage (data not shown), or potential mislabeling during de-identification of specimens. Beyond that, differences in assay design or imprecision may contribute to the discordance observed at the lower end of the AMR.

The high concordance between Alinity m EBV and LDT EBV was also confirmed by the evaluation of the longitudinal data of 24 individual patient courses which showed very similar plasma kinetics. This high level of agreement was unexpected, but it may be attributed to a comparable extraction efficiency and target region/amplicon size of the two assays. Additionally, both assays are standardized to the first WHO IS. In fact, if we take into account this standardization, it alone reduces the inter-lab SD across multiple sample types to <0.5 Log IU/mL (15). Therefore, this finding may not be that unexpected.

Limitations of our study include use of surplus samples for assay comparison resulting in the inability to concurrently test samples with Alinity m and comparator assays. Moreover, in some cases, there was not sufficient sample volume left for additional analysis. In spite of the study limitations, the study demonstrated excellent precision of the Alinity m EBV assay and comparable performance against comparator assays. The observed overall bias between assays was less than 0.5 Log IU/mL and, thus, would not be clinically significant.

Fast result reporting of EBV DNA is especially important for transplant patient management in order to initiate preemptive therapy in case of EBV infection or reactivation. Therefore, already with the current testing procedures using batchwise testing of samples and being performed once or twice a day, the study sites have strived to report EBV DNA loads within 24 h (9) or latest within 72 h in case of weekends. This fast TAT can easily be achieved by the Alinity m system which provides continuous and random access capabilities not requiring batching and allowing testing of all samples upon receipt in the laboratory. A detailed evaluation of how the Alinity m instrument manages random loading and processing of samples for a variety of assays can be found elsewhere (16, 17). This flexibility is combined with a median onboard TAT of less than 3 h as observed in our study. This was achieved despite a total of 369 routine SARS-CoV-2 and CMV study samples being loaded prior or together with the overall 300 EBV study samples, reflecting common testing scenarios in the laboratories. Similar median onboard TATs were also reported in previous studies for other Alinity m assays that compared the Alinity m workflow with a variety of molecular platforms (16, 17).

In conclusion, this study shows that the Alinity m EBV assay performs with high precision and reproducibility, providing accurate quantitation of EBV in verification panel samples and clinical plasma specimens. The Alinity m EBV run on the Alinity m platform enables same-day test result reporting to shorten the time to diagnosis and, thus, to treatment and may improve patient care and outcome.
